# Using magnetoencephalography to investigate brain activity during high frequency deep brain stimulation in a cluster headache patient

**DOI:** 10.2349/biij.3.1.e25

**Published:** 2007-01-01

**Authors:** NJ Ray, ML Kringelbach, N Jenkinson, SLF Owen, P Davies, S Wang, N De Pennington, PC Hansen, J Stein, TZ Aziz

**Affiliations:** 1 Department of Physiology, Anatomy and Genetics, University of Oxford, UK; 2 Department of Neurosurgery, Radcliffe Infirmary, Oxford, UK; 3 Department of Neurology, Radcliffe Infirmary, Oxford, UK.

## Abstract

**Purpose:**

Treatment-resistant cluster headache can be successfully alleviated with deep brain stimulation (DBS) of the posterior hypothalamus [1]. Magnetoencephalography (MEG) is a non-invasive functional imaging technique with both high temporal and high spatial resolution. However, it is not known whether the inherent electromagnetic (EM) noise produced by high frequency DBS is compatible with MEG.

**Materials and methods:**

We used MEG to record brain activity in an asymptomatic cluster headache patient with a DBS implanted in the right posterior hypothalamus while he made small movements during periods of no stimulation, 7 Hz stimulation and 180 Hz stimulation.

**Results:**

We were able to measure brain activity successfully both during low and high frequency stimulation. Analysis of the MEG recordings showed similar activation in motor areas in during the patient’s movements as expected. We also observed similar activations in cortical and subcortical areas that have previously been reported to be associated with pain when the patient’s stimulator was turned on or off [2,3].

**Conclusion:**

These results show that MEG can be used to measure brain activity regardless of the presence of high frequency deep brain stimulation.

## INTRODUCTION

Deep brain stimulation (DBS) offers a unique opportunity to study the underlying pathophysiology of the human disorders that can be treated using DBS. Unfortunately, functional magnetic resonance imaging (fMRI) is inappropriate for mapping DBS-induced changes in brain activity, as the strong magnetic fields involved in fMRI can cause overheating or movement of the electrode or the associated implantable pulse generator that is situated under the skin of the chest. Magnetoencephalography (MEG) records the magnetic component of the electromagnetic signal generated by the brain. It can provide a spatial resolution that rivals fMRI techniques (around 5 mm³), yet unlike fMRI it has a temporal resolution in the order of milliseconds.

The choice of frequency is dependent on the site of stimulation. We have previously shown that it is possible to use MEG to record the brain activity of a patient with a DBS electrode implanted within the periventricular grey/periaquaductal grey to control phantom limb pain [4] with a therapeutic stimulation frequency of 7 Hz.

However, the majority of DBS patients receive high-frequency stimulation (130-180 Hz). It is possible that the significant increase in EM energy generated at high-frequency DBS will interfere with the MEG sensors. Therefore, it is important to assess whether MEG is suitable for studying patients using high frequency settings.

## METHODS

### Case history

The patient, described previously by Owen [11], was a 56-year-old male with an 11-year history of cluster headache attacks. The headaches had a seasonal pattern starting in September or October every year and occurred three to four times a day, lasting for 45 minutes on average. The pain originated over the right forehead and radiated to the ipsilateral vertex and was associated with lacrimation and excess rhinorrhea.

He was previously given carbamazepine, methysergide (2 mg three times daily), cafergot, co-proxamol, verapamil (240 mg twice daily), lithium (800 mg twice daily), amitryptiline and at the time of referral was partially controlled on injections of sumatriptan and high-dose prednisolone.

### Procedure

The patient’s stimulator was turned off for 30 minutes prior to scanning. We attached electrodes to his forearm for an EMG measurement. He was then scanned for 10 minutes. At 22-second intervals he was asked to rate his pain by pressing a button to stop a line moving along a scale measuring from “not painful” to “very painful”. The screen was blank between ratings.

The patient’s stimulator was then turned on and set to 7 Hz. After another 5 minutes we began the second 10-minute scan along with the rating task. The protocol was then repeated a final time with the stimulator set at 180 Hz.

### Data acquisition

The recordings were collected using a 275-channel CTF Omega system (CTF Systems Inc., Port Coquitlam, Canada) at Aston University. Data were sampled at 1200 Hz with an anti-aliasing cut-off filter of 200 Hz.

The patient was scanned with MRI before and after surgery to get a high-resolution T1 volume with 1x1x1 mm voxel dimensions. After the MEG scan, we used a 3-D digitizer (Polhemus Fastrack) to digitize the shape of the patient’s head relative to the position of the headcoils, with respect to the nasion, and the left and right ear, which could be later registered on the MRI scan. There were no significant head movements between conditions.

The EMG was recorded on one of the MEG system’s EEG channels and was later used to identify the motor response to the pain-rating task.

### Image analysis

In line with the previous experiment (Kringelbach, 2006), the data were analysed using Synthetic Aperture Magnetometry (SAM). This is an adaptive beam-forming technique used to analyse EEG and MEG data, which provides continuous 3-D images of cortical power changes [5]. SAM can highlight changes in cortical synchronisation; some of which have been shown to be related to the hemodynamic responses found with fMRI [6].

In SAM, the brain is divided into many target locations (typically into voxels with dimensions of 5x5x5 mm^3^). An optimal spatial filter was computed for each voxel, linking the signal at the target location to the signals recorded at the MEG sensor locations. The filter leaves signals from the location of interest unperturbed whilst signals from other locations are attenuated. This focusing is achieved by selectively weighing the contribution that each sensor makes to the overall output of the spatial filter. The jack-knife statistical method was used to calculate the total amount of power in the specified frequency band within each of the active (during button press) and passive (2 seconds before button press) states to produce a t-map. A 3-D image of the brain was then produced by repeating this procedure for each voxel (5x5x5 mm^3^). Power changes in the 10-20 Hz, 20-30 Hz and 30-60 Hz frequency bands were then calculated between the active and passive states, and the threshold was set at t>2.3.

## RESULTS

**Figure 1:**
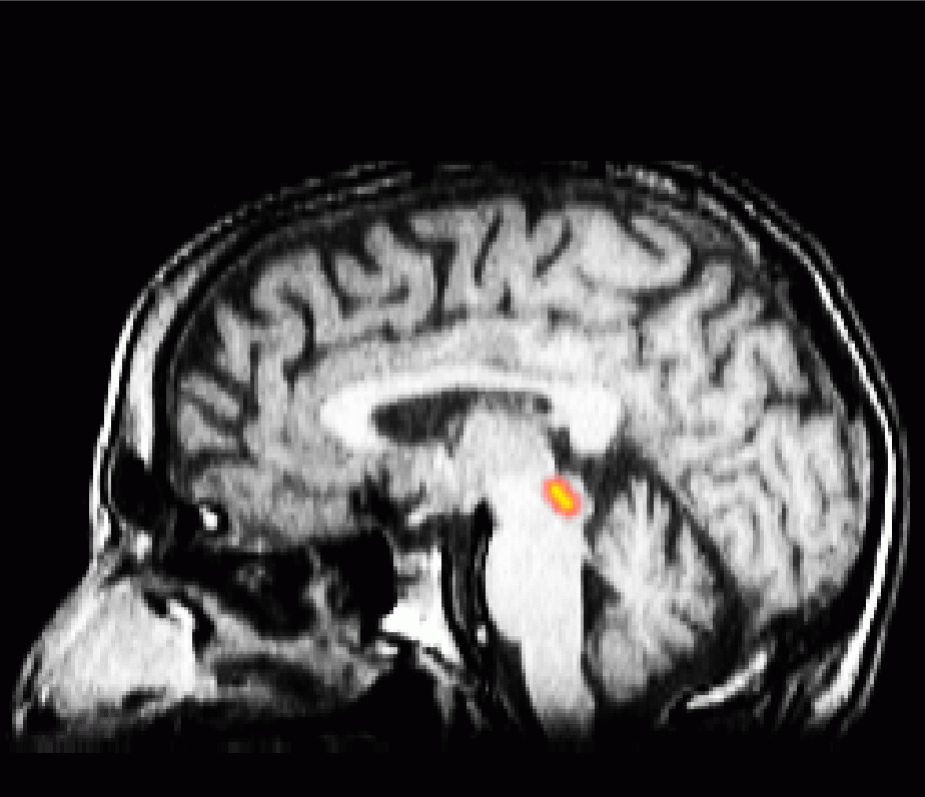
Brainstem activation (PAG) in the 10-20 Hz frequency band when the patient’s stimulator was turned off.

We were able to record the patient’s brain activity with MEG in all conditions regardless of stimulator activity (Table 1). In all conditions, somatosensory and motor cortex were activated in the active (button-press) compared with passive (100 ms before the button-press) states. We also found activation in the 10-20 Hz frequency band in the periaquaductal grey (PAG) only when the patient’s stimulator was turned off (Figure 1).

We did not design our tasks to look for specific activations in any particular region. So we cannot say whether these activations fit with expectations or not. We report them here as they may serve as guidelines for directing future investigations. The activations in PAG in particular must be considered with caution, given the unreliability with which our analysis can localise sources far below the cortex. When we threshold the activations at a t value of 2 (corresponding to a confidence interval of 95%), we found that the activation could be located at around 15 m left and right of the PAG but the peak is at the location we have listed in Table 1

**Figure 2:**
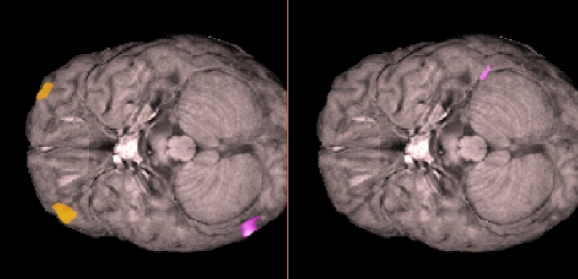
Orbitofrontal activations in the 10-20 Hz frequency band during 180 Hz stimulation (left image), but not during no stimulation (right image).

When the stimulator was turned on, the fMRI showed activations in frontal brain regions previously associated with the pain relief network (Figure 2).

**Figure 3:**
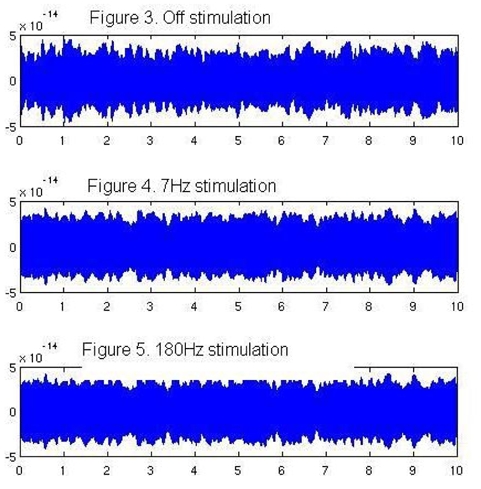
Raw data from a central channel during no stimulation, and with stimulation at 7 Hz and 180 Hz.

Figures 3-5 show 10 seconds of raw data traces in all three conditions.

## DISCUSSION

We have shown that it is possible to use MEG to study changes in brain activity even during high-frequency DBS. In all conditions, we found activity in brain regions serving motor control when the patient was pressing a button to rate his pain. While this is not a new finding in itself, it does show that it is feasible to use MEG and synthetic aperture magnetometry (SAM) techniques to image the brain during high-frequency DBS. However, we do not know yet if the stimulator has produced artefacts that are not detectable with the analysis we have carried out. It is possible that the sensor data is compromised by the presence of the battery, electrode and the stimulation itself, but it is undetectable after the filtering is done in SAM. Further work is needed to ascertain whether different types of analysis will still yield data resistant to artefacts from DBS. This work must also use multiple subject numbers to ensure that the findings presented here are reliable.

This study was intended to investigate the effects of high-frequency stimulation on MEG recordings. During analysis, however, we found patterns of activation that could be explained by the current opinion on pain and pain-relief. The posterior hypothalamus contains several neurochemically distinct cell groups. One of these is the Hypocr/Orx neurons that are activated by nociceptive stimuli and reach structures involved in nociceptive relay and modulation, including the PAG [7]. We found PAG activation only when the patient’s stimulator was turned off, which may be related to these connections. In a fMRI study, it was found that PAG was activated if subjects were anticipating a painful stimulus, even before they were subjected to pain [2]. The patient in the present study was aware that his stimulator had been turned off. The activations in PAG may have been related to the patient anticipating his pain to return.

We also found activations that have been associated with the pain relief functions of the mid-anterior orbitofrontal cortex [6], which were most effective in the 180 Hz stimulation condition. This result is similar to that reported in a previous paper studying the effects of low-frequency DBS [4].

As well as treating the motor and pain conditions previously mentioned, DBS is being applied to an extending number of disorders; OCD [8], depression [9], epilepsy [10]. However, the mechanisms by which DBS is effective in any of these situations is unclear. Although we must proceed with caution when considering our activations, particularly in PAG given that it is sub-cortical, this study suggests that MEG will be a useful tool for understanding the neural changes induced by DBS.

## ACKNOWLEDGEMENT

Charles Wolfson Charitable Trust, Norman Collisson Foundation, Templeton Foundation and MRC.
